# Exploring non-autonomous protein homeostasis driven by glutamatergic neurons

**DOI:** 10.17912/micropub.biology.002119

**Published:** 2026-05-20

**Authors:** Adam J Hruby, Aeowynn J Coakley, Evan Dittus, Andrew Bong, Camilla Pearson, Jing Wang, Peter J Mullen, Gilberto Garcia, Ryo Higuchi-Sanabria

**Affiliations:** 1 Leonard Davis School of Gerontology, University of Southern California; 2 Immunology and Immune Therapeutics, University of Southern California; 3 Norris Comprehensive Cancer Center, Keck School of Medicine University of Southern California

## Abstract

Neuronal overexpression of *xbp-1 s* , a regulator of the endoplasmic reticulum unfolded protein response (UPR ^ER^ ), induces non-autonomous UPR ^ER^ activation in distal tissues of *Caenorhabditis elegans* . Specific neuronal subtypes, including glutamatergic, octopaminergic, and GABAergic neurons, have been implicated in enhancing intestinal proteostasis. Here, we investigated the mechanisms underlying this effect. Glutamatergic *xbp-1 s* mediated proteostasis improvement was independent of endogenous *xbp-1* but required the transcription factor HLH-30 , potentially via autophagy and ER-associated degradation pathways. In contrast, octopaminergic and GABAergic signaling yielded limited insight. These findings highlight the complexity of neuronal control of organismal proteostasis through non-autonomous UPR ^ER^ signaling pathways.

**Figure 1. Investigating the mechanisms behind glutamatergic, octopaminergic, and GABAergic non-autonomous xbp-1s signaling-mediated proteostasis:** For **(A-D)** , animals were imaged on day 1, 5, and 9 of adulthood. Experiments were performed across 3 biological replicates with 2 technical replicates each, for a total of 6 replicates. **&nbsp;(A)** Quantification of protein foci within animals expressing intestinal polyglutamine 44 repeats ( *vha-6p::polyQ44::YFP* ) in control or glutamatergic ( *eat-4 p* ) *xbp-1 s* animals with and without a nonsense mutation in *xbp-1* , *xbp-1 ( zc12* ). **(B)** Quantification of protein foci within animals expressing intestinal polyglutamine 44 repeats ( *vha-6p::polyQ44::YFP* ) in control or glutamatergic ( *eat-4 p* ) *xbp-1 s* animals with and without loss-of-function *hlh-30 ( tm1978* ) mutation. **(C)** Quantification of protein foci within animals expressing intestinal polyglutamine 44 repeats ( *vha-6p::polyQ44::YFP* ) in control or glutamatergic ( *eat-4 p* ) *xbp-1 s* animals with and without mutation blocking glutamatergic signaling, *eat-4 ( ky5* ). **(D)** Quantification of protein foci within animals expressing intestinal polyglutamine 44 repeats ( *vha-6p::polyQ44::YFP* ) in control or glutamatergic ( *eat-4 p* ) *xbp-1 s* animals with and without mutation blocking serotonergic signaling, *tph-1 ( mg280* ). **(E)** Selected protein degradation gene ontology terms of differentially expressed genes in whole body upon *xbp-1 s* overexpression in glutamatergic ( *eat-4 p* ), octopaminergic ( *tbh-1 p* ), or GABAergic ( *unc-25 p* ) neurons. Analysis was performed using WormEnrichr. **(F)** Representative images of animals expressing intestinal polyglutamine 44 repeats ( *vha-6p::polyQ44::YFP* ) in glutamatergic ( *eat-4 p* ) *xbp-1 s* animals under RNAi-mediated knockdown of potential mediators of improved proteostasis. Animals were imaged on day 5 of adulthood. Scale bar represents 500 µm. **(G)** Quantification of protein foci within animals expressing intestinal polyglutamine 44 repeats ( *vha-6p::polyQ44::YFP* ) in control, glutamatergic ( *eat-4 p* ), octopaminergic ( *tbh-1 p* ), or GABAergic ( *unc-25 p* ) *xbp-1 s* animals under RNAi-mediated knockdown of potential mediators of improved proteostasis. Comparisons were made between empty vector and RNAi conditions within each strain. All significant differences are indicated with a star. Animals were imaged on day 5 of adulthood, and experiments were performed across 3 biological replicates. **(H)** Quantification in (G) is represented as the ratio of polyQ foci in glutamatergic ( *eat-4 p* ), octopaminergic ( *tbh-1 p* ), or GABAergic ( *unc-25 p* ) *xbp-1 s* animals relative to the matched N2 control condition for each RNAi treatment. Ratios were calculated by dividing the average polyQ foci number in the *xbp-1 s* overexpression group by the mean polyQ foci number in the corresponding N2 group within the same RNAi condition. ns = p > 0.05, * = p ≤ 0.05, ** = p < 0.01, *** = p <0.001, **** = p ≤ 0.0001. For A-G, A Mann-Whitney test was used to determine significance. For H, a Shapiro-Wilk test was used to determine normality and an unpaired t-test was used to determine significance.

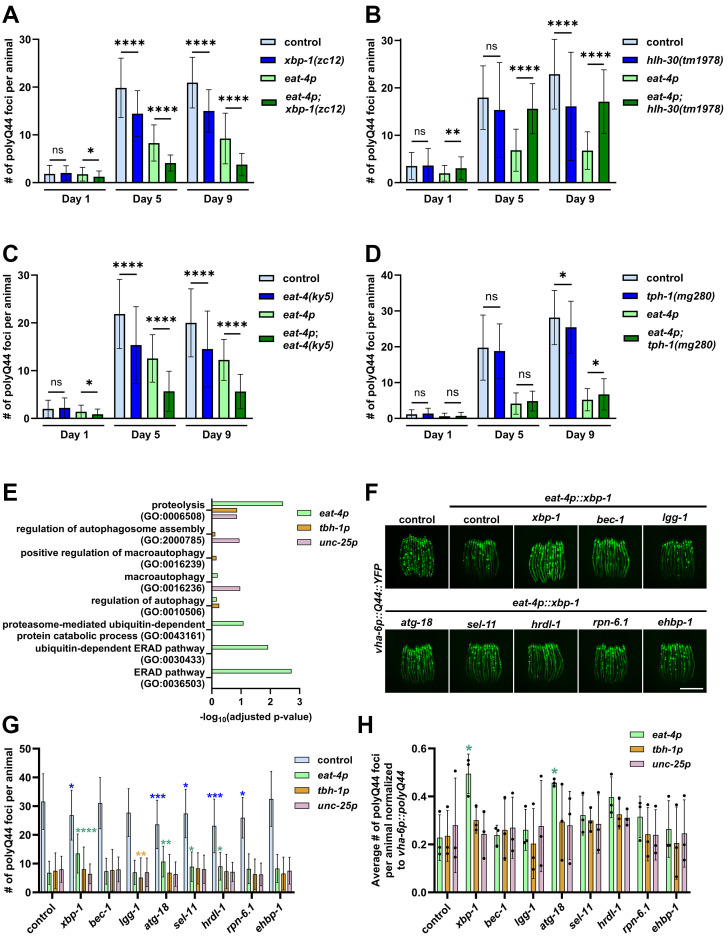



## Description

Restoration of cellular homeostasis after stress is essential for maintaining cellular function and overall organismal health. Stress affecting specific organelles can trigger adaptive responses that restore proper homeostatic states. One such pathway is the unfolded protein response of the endoplasmic reticulum (UPR ^ER^ ) which is regulated by three distinct branches, of which the most well-studied is the IRE-1 / XBP-1 pathway. Inositol-requiring enzyme 1 ( IRE-1 ) is an ER-membrane protein that responds to ER stress by dimerizing and undergoing autophosphorylation, thereby activating its RNAse domain. This results in splicing of the mRNA encoding the transcription factor X-box binding protein ( *xbp-1* ) into its active *xbp-1 s* (spliced) form. XBP-1 s regulates expression of genes involved in ER quality control including protein chaperones, lipid metabolism, autophagy, ER-associated degradation (ERAD), and the ubiquitin proteosome system (UPS) (Dutta et al., 2022).

When *xbp-1 s* is ectopically expressed in neurons of the nematode, *Caenorhabditis elegans* , the UPR ^ER^ is activated in distal tissues via a non-autonomous stress signal (Taylor & Dillin, 2013). This neuron-to-body signal is mediated by a diverse array of neuronal subtypes, including serotonergic and dopaminergic neurons (Higuchi-Sanabria et al., 2020), tyraminergic neurons (Özbey et al., 2020), and glutamatergic, octopaminergic, and GABAergic neurons (Coakley, Hruby et al., 2025). Recently published work found that *xbp-1 s* overexpression in glutamatergic neurons (driven by the *eat-4* promoter), and to a lesser degree in octopaminergic neurons (driven by the *tbh-1* promoter) and GABAergic neurons (driven by the *unc-25* promoter) improves the proteostatic capacity of the intestine (Coakley, Hruby et al., 2025). However, the mechanisms by which these neurons signal to the periphery and what proteostatic pathways are induced by this signal has yet to be explored.

To investigate how these neuronal subtypes signal to distal tissue to improve proteostasis, we previously utilized transgenic strains with *xbp-1 s* overexpression in glutamatergic, octopaminergic, and GABAergic neurons (referred to as glutamatergic, octopaminergic, or GABAergic *xbp-1 s* for simplicity) crossed with strains expressing fluorescently-labeled polyglutamine 44 repeats in the intestine (referred to as polyQ44). PolyQ44 forms insoluble protein foci (Morley et al., 2002) whose abundance serves as a proxy for assessing proteostatic capacity. Glutamatergic, octopaminergic, and GABAergic *xbp-1 s* overexpression was found to reduce the number of polyQ44 foci, suggesting improved proteostasis (Coakley, Hruby et al., 2025).

Since glutamatergic *xbp-1 s* animals displayed the largest changes (Coakley, Hruby et al., 2025), we sought to further explore the mechanism driving the improved proteostasis in these animals. First, we aimed to determine whether *xbp-1* was required in peripheral tissues, as previous studies have shown that neuronal *xbp-1 s* activates peripheral *xbp-1* to promote its beneficial effects. Therefore, we crossed glutamatergic *xbp-1 s* polyQ44 expressing animals into the *xbp-1 ( zc12 )* mutant, which contains a nonsense mutation in *xbp-1* (Calfon et al., 2002). Surprisingly, improved polyQ44 clearance was not dependent on peripheral *xbp-1* , as the *xbp-1 ( zc12 )* mutant failed to suppress the reduction in polyQ44 foci in glutamatergic *xbp-1 s* animals ( **Fig. 1A** ). Non-autonomous UPR ^ER^ signaling from both neurons (Imanikia et al., 2019) and glia (Metcalf et al., 2024) have been shown to activate HLH-30 , the *C. elegans* ortholog of Transcription Factor EB (TFEB), in peripheral tissues to enhance lysosomal function and improved autophagy. Therefore, to determine whether glutamatergic *xbp-1 s* similarly activates HLH-30 to promote peripheral proteostasis, we crossed glutamatergic *xbp-1 s* polyQ44 expressing animals into the loss-of-function *hlh-30 ( tm1978 )* mutant strain (Settembre et al., 2013). We found that HLH-30 is indeed required for the improvement in peripheral proteostasis driven by glutamatergic *xbp-1* ( **Fig. 1B** ).

To continue to dissect the neuronal signaling required for the improved proteostasis of glutamatergic *xbp-1 s* animals, we crossed this strain into an *eat-4 ( ky5 )* mutant strain which prevents glutamatergic signaling (Lee et al., 1999). Surprisingly, the *eat-4 ( ky5 )* mutation did not suppress the reduction of polyQ44 foci in glutamatergic *xbp-1 s* animals ( **Fig. 1C** ), suggesting the improvement in proteostasis does not require glutamatergic signaling. The *eat-4* promoter drives *xbp-1 s* expression in 79 neurons, some of which are not exclusively glutamatergic (Loer & Rand, 2022). Therefore, improvement in proteostasis may be driven by a subset of these neurons, potentially through *xbp-1 s* overexpression in serotonergic neurons which was previously demonstrated to drive chaperone induction in a non-autonomous fashion (Higuchi-Sanabria et al., 2020). Neurons reported to be both glutamatergic and serotonergic in hermaphrodites include: AIM, ASG, and I5 (Loer & Rand, 2022). To investigate the contribution serotonergic signaling plays in glutamatergic *xbp-1* -mediated proteostasis, we crossed glutamatergic *xbp-1 s* polyQ44 expressing animals into a *tph-1 ( mg280 )* mutant strain which blocks serotonergic signaling (Sze et al., 2000). Only a very minor increase in polyQ44 foci was observed at day 9 of adulthood in the *tph-1 ( mg280 )* mutant animals expressing glutamatergic *xbp-1 s* , suggesting that serotonergic signaling does not play a major role in glutamatergic *xbp-1* -mediated improvements in peripheral proteostasis ( **Fig. 1D** ). Thus, the signal mediating this effect remains to be determined. Future studies specifically overexpressing *xbp-1 s* in subpopulations of glutamatergic neurons (e.g., AIM, ASG, and I5) could prove fruitful for further dissection of non-autonomous *xbp-1 s* -mediated proteostasis.

The IRE-1 / XBP-1 pathway is known to activate several different processes which have the potential to resolve proteotoxic stress, including autophagy, ERAD, and the UPS. To determine which of these processes are required for the improvement in proteostasis, we first analyzed the transcriptome of glutamatergic, octopaminergic, and GABAergic *xbp-1 s* animals using previously published bulk RNA sequencing data (Coakley, Hruby et al., 2025). Gene ontology analysis of the differentially expressed genes revealed terms associated with proteostatic pathways ( **Fig. 1E** ). In particular, glutamatergic *xbp-1 s* was associated with terms related to ERAD, while octopaminergic and GABAergic *xbp-1 s* was associated with terms related to autophagy ( **Fig. 1E** ). To test the dependency of improved proteostasis on these processes, we performed an RNA interference (RNAi) screen. Genes involved in the following processes were knocked down: IRE-1 / XBP-1 ( *xbp-1* ), autophagy ( *bec-1* , *lgg-1* , *atg-18* ; Chen et al., 2017), ERAD ( *sel-11* , *hrdl-1* ; Sasagawa et al., 2007), the UPS ( *rpn-6.1* ; Vilchez et al., 2012), and lipophagy ( *ehbp-1* ; Shi et al., 2010). Representative images for glutamatergic *xbp-1 s* animals are shown in **Fig. 1F** . Quantification of polyQ44 foci revealed several hits ( **Fig. 1G- H** ). Knockdown of *xbp-1 s* , *atg-18* , *sel-11* , and *hrdl-1* increased the number of polyQ44 foci in the glutamatergic *xbp-1 s* animals, although not to the same levels as the control ( **Fig. 1G** ). This suggests the potential for both autophagy and ERAD to be involved in glutamatergic *xbp-1 s* -mediated clearance of polyQ44 foci, mirroring the RNA sequencing data ( **Fig. 1E** ). The requirement for HLH-30 , a known regulator of autophagy in *C. elegans* (Lapierre et al., 2013), in glutamatergic *xbp-1 s* -mediated improved proteostasis ( **Fig. 1B** ) provides further evidence that autophagy is involved in this process. In contrast, knockdown of autophagy, ERAD, the UPS, or lipophagy components did not affect the number of polyQ44 foci in octopaminergic or GABAergic *xbp-1 s* animals. In addition, *lgg-1* knockdown resulted in a mild, but statistically significant, reduction in polyQ44 foci in octopaminergic *xbp-1 s* animals. Only knockdown of *xbp-1* and *atg-18* in glutamatergic *xbp-1 s* overexpressing animals were found to significantly increase polyQ44 foci in the normalized data ( **Fig. 1H** ). Trends for increased polyQ44 foci were present under *sel-11* and *hrdl-1* knockdown, although these were not statistically significant. No significant differences in normalized polyQ44 foci counts were found in octopaminergic and GABAergic xbp-1 s overexpressing animals. These data suggest that glutamatergic *xbp-1 s* promotes proteostasis potentially via more canonical pathways including autophagy and potentially ERAD, while octopaminergic and GABAergic neurons employ other pathways.

Overall, this study further elucidates the pathways through which glutamatergic, octopaminergic, and GABAergic *xbp-1 s* improve proteostasis. We find that while glutamatergic *xbp-1 s* animals utilize canonical proteostasis mechanisms to promote protein processing, octopaminergic and GABAergic neurons do not. Surprisingly, the improvement in proteostasis found in glutamatergic *xbp-1 s* animals did not require a canonical XBP-1 dependent mechanism in the periphery nor glutamatergic or serotonergic signaling. However, the suppression of HLH-30 did block improved proteostasis, suggesting peripheral activation of HLH-30 is a likely mediator. This study has several limitations: first, we utilize the polyQ44 animal as a proxy for proteostasis; however, this is an artificial system that may not always recapitulate endogenous proteostasis pathways. Additionally, we focused on glutamatergic signaling of our *eat-4 p:: xbp-1 s* animals, although the *eat-4* promoter is expressed in several neuronal subtypes. Despite these caveats, our data align well with our transcriptomic analysis and revealed the potential for non-autonomous glutamatergic *xbp-1 s* signaling to be activating ERAD as well as autophagy through HLH-30 to clear polyQ44 foci. This work furthers our understanding of the potential mechanisms whereby non-autonomous *xbp-1 s* signaling drives proteostasis.

## Methods

*C. elegans* maintenance

*C. elegans* were maintained on standard nematode growth medium (NGM) plates fed with OP50 *E. coli* B strain at 15°C. All strains used in this study are viable across the 15–20°C range; however, stocks were maintained at 15°C to slow population growth and reduce the frequency of passaging, thereby minimizing genetic drift. Experimental assays were conducted at 20°C. Standard NGM plates contained the following: Bacto-Agar (Difco) 2% w/v, Bacto Peptone 0.25% w/v, NaCl _2_ 0.3% w/v, 1 mM CaCl _2_ , 5 µg/ml cholesterol, 0.625 mM KPO _4_ pH 6.0, 1 mM MgSO _4_ . Animals were bleached and L1 arrested to synchronize age. Briefly, worms were collected into a 15 mL conical tube using M9 solution (22 mM KH _2_ PO _4_ monobasic, 42.3 mM NaHPO _4_ , 85.6 mM NaCl, 1 mM MgSO _4_ ) and exposed to a bleaching solution (1.8% sodium hypochlorite, 0.375 M NaOH in M9) until complete disintegration of carcasses. Intact eggs were then washed four times with M9 solution by centrifugation at 1,100 x g for 30 seconds. After the final wash, animals were L1 arrested by incubating overnight in M9 at 20°C on a rotator for a maximum of 24 hours. After arresting, worms were transferred to growth conditions at 20°C utilizing HT115 *E. coli* K strain carrying an empty pL4440 vector, referred to as empty vector (EV), for all experiments. NGM plates for experimental conditions contained the following: Bacto-Agar (Difco) 2% w/v, Bacto Peptone 0.25% w/v, NaCl _2_ 0.3% w/v, 1 mM CaCl _2_ , 5 µg/ml cholesterol, 0.625 mM KPO _4_ pH 6.0, 1 mM MgSO _4_ , 100 µg/mL carbenicillin, 1 mM IPTG. All experiments were performed using worms grown at 20°C. For all aging experiments, 100 μL of 10 mg/mL (+)-5-Fluorodeoxyuridine (FUDR) was placed directly on the bacterial lawn and worms were moved onto FUDR-containing plates on day 1 of adulthood. For all experiments including *hlh-30 ( tm1978* ) mutants, animals were not L1 arrested as HLH-30 is necessary for survival during starvation (Murphy et al., 2019); after bleaching, eggs were placed directly onto experimental plates.

Stereoscope imaging

For whole-worm imaging, synchronized animals were grown on experimental NGM plates seeded with EV bacteria or RNAi bacteria. Animals were imaged on day 1 of adulthood and for aging experiments also imaged on day 5 and day 9 of adulthood. To induce paralysis, 13 worms were placed in a drop of 100 mM sodium azide in M9 on standard NGM plates without bacteria. Paralyzed animals were then lined up alongside each other and imaged on a Leica M205FCA automated fluorescent stereomicroscope running LAS X software and equipped with a standard GFP filter, Leica LED3 light source, and Leica K5 camera. For all imaging experiments, at least 3 biological replicates were performed with 2 technical replicates each, aside from **Fig. 1G** in which only 1 technical replicate per biological replicate was performed. *vha-6p::Q44::YFP* quantification was performed by counting the number of foci in individual worms using ImageJ Fiji (Schindelin et al., 2012), and statistical analysis was performed with GraphPad Prism 10 software using a Mann-Whitney test.

Statistics and reproducibility

All statistical analyses were performed using GraphPad Prism 10 software. No assumptions were made about data distribution, and p-values less than 0.05 were considered significant. For all experiments, a Mann-Whitney test was used to determine significance, aside from **Fig. 1H** in which a Shapiro-Wilk test was used to determine normality and an unpaired t-test used to determine significance. At least 3 biological replicates were performed for each experiment. Representative images from **Fig. 1A- D** are contained in **Extended Data 1** and all raw data is contained in **Extended Data 2** . RNA-seq analysis was performed using a previously published dataset (Coakley, Hruby et al., 2025) which is available through Annotare 2.0 ArrayExpress Accession E-MTAB-14132. Gene ontology analysis was performed using WormEnrichr (Chen et al., 2013; Kuleshove et al., 2016).

**Extended Data 1. Representative images for Figure 1A- D:** Representative images for all experiments in Figure 1 are provided as extended data for data transparency. **(A)** Representative images of protein foci within animals expressing intestinal polyglutamine 44 repeats ( *vha-6p::polyQ44::YFP* ) in control or glutamatergic ( *eat-4 p* ) *xbp-1 s* animals with and without a nonsense mutation in *xbp-1* , *xbp-1 ( zc12* ). **(B)** Representative images of protein foci within animals expressing intestinal polyglutamine 44 repeats ( *vha-6p::polyQ44::YFP* ) in control or glutamatergic ( *eat-4 p* ) *xbp-1 s* animals with and without loss-of-function *hlh-30 ( tm1978* ) mutation. **(C)** Representative images of protein foci within animals expressing intestinal polyglutamine 44 repeats ( *vha-6p::polyQ44::YFP* ) in control or glutamatergic ( *eat-4 p* ) *xbp-1 s* animals with and without mutation blocking glutamatergic signaling, *eat-4 ( ky5* ). Animals were imaged on days 1, 5, and 9 of adulthood. **(D)** Representative images of protein foci within animals expressing intestinal polyglutamine 44 repeats ( *vha-6p::polyQ44::YFP* ) in control or glutamatergic ( *eat-4 p* ) *xbp-1 s* animals with and without mutation blocking serotonergic signaling, *tph-1 ( mg280* ). For all panels animals were imaged on days 1, 5, and 9 of adulthood, and images were captured using a Leica M205 stereo microscope. Scale bar represents 500 µm.

## Reagents

**Table:** 

***C. elegans* strain **	**Genotype**	**Source**
Bristol ( N2 )	Wild type	*Caenorhabditis* Genetics Center
MAH602	*sqIs61 [vha-6p::Q44::YFP + rol-6 ( su1006 )] *	Hansen Lab
RHS157	*sybIs3970 [ unc-25 p:: xbp-1 s, myo-2p::GFP]; sqIs61 [vha-6p::Q44::YFP + rol-6 ( su1006 )] *	Sanabria Lab
RHS158	*sybIs3923 [ eat-4 p:: xbp-1 s, myo-2p::mCherry]; sqIs61 [vha-6p::Q44::YFP + rol-6 ( su1006 )] *	Sanabria Lab
RHS161	*sybIs3954 [ tbh-1 p:: xbp-1 s, myo-2p::mCherry]; sqIs61 [vha-6p::Q44::YFP + rol-6 ( su1006 )] *	Sanabria Lab
RHS268	*eat-4 ( ky5 ) III; sqIs61 [vha-6p::Q44::YFP + rol-6 ( su1006 )] *	Sanabria Lab
RHS269	*eat-4 ( ky5 ) III; sybIs3923 [ eat-4 p:: xbp-1 s, myo-2p::mCherry]; sqIs61 [vha-6p::Q44::YFP + rol-6 ( su1006 )] *	Sanabria Lab
RHS292	*xbp-1 ( zc12 ) III; sqIs61 [vha-6p::Q44::YFP + rol-6 ( su1006 )] *	Sanabria Lab
RHS293	*xbp-1 ( zc12 ) III; sybIs3923 [ eat-4 p:: xbp-1 s, myo-2p::mCherry]; sqIs61 [vha-6p::Q44::YFP + rol-6 ( su1006 )] *	Sanabria Lab
RHS338	*tph-1 ( mg280 ) II; sqIs61 [vha-6p::Q44::YFP + rol-6 ( su1006 )] *	Sanabria Lab
RHS339	*tph-1 ( mg280 ) II; sybIs3923 [ eat-4 p:: xbp-1 s, myo-2p::mCherry]; sqIs61 [vha-6p::Q44::YFP + rol-6 ( su1006 )] *	Sanabria Lab
RHS340	*hlh-30 ( tm1978 ) IV; sqIs61 [vha-6p::Q44::YFP + rol-6 ( su1006 )] *	Sanabria Lab
RHS341	*hlh-30 ( tm1978 ) IV; sybIs3923 [ eat-4 p:: xbp-1 s, myo-2p::mCherry]; sqIs61 [vha-6p::Q44::YFP + rol-6 ( su1006 )] *	Sanabria Lab

## Data Availability

Description: Extended Data 1: Figure 1A-D representative images.. Resource Type: Image. DOI: https://doi.org/10.22002/24qdk-rzg91 Description: Extended Data 2: All raw data.. Resource Type: Dataset. DOI: https://doi.org/10.22002/jvnja-xj859
